# Subjective well-being in patients with pemphigus: a path analysis

**DOI:** 10.1007/s10198-019-01067-w

**Published:** 2019-05-16

**Authors:** Ariel Mitev, Fanni Rencz, Béla Tamási, Krisztina Hajdu, Márta Péntek, László Gulácsi, Andrea Szegedi, Zsuzsanna Bata-Csörgő, Ágnes Kinyó, Miklós Sárdy, Valentin Brodszky

**Affiliations:** 1 Department of Marketing, Corvinus University of Budapest, Fővám tér 8, Budapest, 1093 Hungary; 2 Department of Health Economics, Corvinus University of Budapest, Fővám tér 8, Budapest, 1093 Hungary; 3 Hungarian Academy of Sciences, Premium Postdoctoral Research Program, Nádor u. 7, Budapest, 1051 Hungary; 4 Department of Dermatology, Venereology and Dermatooncology, Faculty of Medicine, Semmelweis University, Mária u. 41, Budapest, 1085 Hungary; 5 Departments of Dermatology, University of Debrecen, Nagyerdei krt. 98, Debrecen, 4032 Hungary; 6 Department of Dermatological Allergology, University of Debrecen, Nagyerdei krt. 98, Debrecen, 4032 Hungary; 7 Department of Dermatology and Allergology, Albert Szent-Györgyi Medical Centre, University of Szeged, Korányi fasor 6, Szeged, 6720 Hungary; 8 Department of Dermatology, Venereology and Oncodermatology, University of Pécs Medical School, Akác u. 1, Pécs, 7632 Hungary

**Keywords:** Pemphigus vulgaris, Pemphigus foliaceus, Path analysis, Health-related quality of life, Satisfaction with life, Subjective well-being, I10

## Abstract

**Background:**

Pemphigus is a chronic autoimmune blistering disease of the skin and mucosa severely impairing patients’ health-related quality of life (HRQoL). To date, no studies have measured subjective well-being in terms of life satisfaction in pemphigus. Our main objective was to evaluate satisfaction with life in patients with pemphigus, and to analyse its relationship with clinical severity and HRQoL.

**Methods:**

A cross-sectional survey was carried out enrolling 77 patients with pemphigus. Subjective well-being was measured using the Satisfaction with Life Scale (SWLS). HRQoL was assessed by the Dermatology Life Quality Index (DLQI) and EQ-5D-5L. Disease severity was measured by Autoimmune Bullous Skin Disorder Intensity Score (ABSIS).

**Results:**

Mean ABSIS, DLQI, EQ-5D-5L and SWLS scores of patients were 11.7 (SD 17.3), 5.4 (6.8), 0.84 (0.22) and 4.76 (SD 1.52), respectively. The proportion of patients indicating extreme dissatisfaction, dissatisfaction, slightly below average in life satisfaction, average satisfaction, high satisfaction and very high satisfaction with life was 6 (7.8%), 5 (6.5%), 14 (18.2%), 16 (20.8%), 21 (27.3%) and 15 (19.5%), respectively. Life satisfaction was independent from age, gender, level of education and type of disease. A path analysis revealed that there was no direct relationship between ABSIS and SWLS (beta = − 0.09; *p* = 0.428); however, the following indirect path was confirmed: ABSIS → DLQI → EQ-5D-5L → SWLS.

**Conclusions:**

Disease severity and HRQoL measures regularly used to assess patients’ health status may be complemented with a measure of subjective well-being, such as SWLS, to achieve a more holistic assessment of patients’ lives and optimise pemphigus care.

## Introduction

Pemphigus is a rare, autoimmune blistering disease with two major subtypes pemphigus vulgaris and pemphigus foliaceus [1]. Pemphigus vulgaris accounts for approximately 70–90% of all cases with an annual incidence rate of 0.76–32 per million [2, 3]. Mean age of onset is usually between 50 and 60 years. Pemphigus causes blisters and erosions developing on the skin or mucous membranes, such as in the mouth. Physical symptoms include itching, skin burning and pain. Pemphigus has a strong influence on patients’ health-related quality of life (HRQoL), but it largely affects other aspects of patients’ life, such as social activities or productivity [4–6]. It has been shown that pemphigus is associated with one of the largest HRQoL impairment among chronic skin diseases [4, 5, 7–9].

Recently, subjective well-being has gained increasing attention in health policy. Subjective well-being reflects an overall evaluation of the quality of a person’s life from their own perspective [10]. Evidence on subjective measures of well-being is in the focus of interest to provide a basis for reimbursement decisions about health technologies [11]. To use well-being outcomes in resource allocation decisions, the relationship between subjective well-being and the different dimensions of health, such as clinical symptoms, disease-specific and general HRQoL, needs to be explored [12]. Life satisfaction is a core indicator of people’s subjective well-being. Satisfaction with life can be used as an outcome measure, as it is directly related to HRQoL. In patients suffering from chronic diseases, both physical and mental problems may have a substantial negative impact on satisfaction with life [13].

Patients’ interpersonal relationships, social activities, sexual life and self-esteem are commonly adversely affected by chronic dermatological diseases [14]. In addition, patients may be dissatisfied with treatments and healthcare in general. Unemployment rate was also found higher in chronic skin diseases compared to health controls, and a proportion of patients also face a lower earning potential [15–18]. Dissatisfaction with life has been earlier reported in patients with atopic dermatitis, psoriasis, hidradenitis suppurativa, melanoma, systemic lupus erythematosus and urticaria [19–25]. Life satisfaction, however, has not yet been measured in pemphigus patients. Therefore, our main objective was to evaluate the satisfaction with life in patients with pemphigus and to analyse the relationship between severity of clinical symptoms, HRQoL and life satisfaction as an indicator of subjective well-being.

## Methods

### Study population and setting

Between December 2014 and June 2017, a cross-sectional study was conducted in four academic dermatology departments in Hungary. Consecutive patients over 18 years of age diagnosed with any form of pemphigus were enrolled after an informed consent form was read and signed. Permission for conducting the study was granted by the National Scientific and Ethical Committee (reference No. ETT-TUKEB 27416-3/2016/EKU).

First part of the survey asked patients about demographics, employment status, self-reported disease severity, utilisation of healthcare services in the past 12 months, HRQoL, and satisfaction with life. Dermatologists filled in the second part of the questionnaire. Based on medical records they provided data on clinical characteristics, medications, medical history and disease severity.

### Outcome measures

#### EQ-5D-5L

EQ-5D-5L is a widely used generic instrument to assess general HRQoL [26, 27]. It consists of two parts: a descriptive system and a visual analogue scale (EQ VAS). The descriptive system involves the following five dimensions of general HRQoL: mobility, self-care, usual activities, pain/discomfort and anxiety/depression. For each dimension, patients may choose from five response levels (no problems = 1, slight problems = 2, moderate problems = 3, severe problems = 4 and unable to/extreme problems = 5) resulting in a total of 3125 (5^5^) unique health states. In the absence of a national value set in Hungary, the value set for England developed by Devlin et al. was applied in this study to derive EQ-5D index scores [28]. Thus, index scores may range from -0.285 to + 1, where − 0.285 corresponds to the worst and +1 to the best possible HRQoL. The second part of the instrument, EQ VAS is a 20-cm long, vertical visual analogue scale with endpoints of ‘0’ (the worst health you can imagine) and ‘100’ (the best you can imagine) recording patients’ self-rating of their overall health.

#### DLQI

Dermatology Life Quality Index (DLQI) is a skin-specific self-reported questionnaire [29, 30]. It consists of ten items covering the common problems affecting HRQoL of patients with skin diseases, such as symptoms, side effects of treatment, daily activities, work or school, personal relationships, leisure activities, and feelings of embarrassment. Each item is scored on a 4-point scale: ‘not at all’ or ‘not relevant’ = 0, ‘a little’ = 1, ‘a lot’ = 2 and ‘very much’ = 3. DLQI total score is calculated by summing the score of each question resulting in a maximum of 30 and a minimum of 0, where the higher the score, the more HRQoL is impaired.

#### SWLS

The Satisfaction with Life Scale (SWLS) is an extensively used questionnaire to quantify people’s global judgement on their subjective well-being [31]. The Hungarian version of SWLS showed a good internal consistency reliability (Cronbach’s *α* 0.84), convergent validity with the Rosenberg Self-esteem Scale and the Purpose in Life Test and an excellent test–retest reliability [32]. SWLS is a five-item instrument each of which is rated on a 7-point scale ranging from ‘strongly disagree’ to ‘strongly agree’. The total score was estimated as the average of the five items resulting in a score between 1 and 7, where a higher score reflects a greater life satisfaction for the individual [31]. The following cut-off scores were used to classify patients into SWLS groups: 1–2 (‘extremely dissatisfied’), 2–3 (‘dissatisfied’), 3–4 (‘slightly below average satisfaction’), 4–5 (‘average satisfaction’), 5–6 (’satisfied’) and 6–7 (’highly satisfied’) [33].

#### ABSIS

Severity of pemphigus was graded using the Autoimmune Bullous Skin Disorder Intensity Score (ABSIS) [34–36]. The ABSIS total score ranges between 0 and 206, where a higher score indicates more severe disease. Subgroups of disease severity were defined based on the following cut-off values: limited (ABSIS 0–3), moderate (ABSIS 4–16), significant (ABSIS 17–52) and extensive (ABSIS 53–206) pemphigus [37].

### Statistical analysis

First, sample and SWLS item characteristics were computed. As a preliminary analysis, Pearson’s correlation coefficients were calculated to explore the relationships between the outcome measures. A correlation coefficient of 0–0.39 was defined as weak, 0.40–0.79 as moderate, and 0.80–1 as a strong correlation. The differences in SWLS scores between subgroups of patients were compared using Mann–Whitney *U* test.

We conducted a path analysis to test a hypothesized model predicting SWLS scores from EQ-5D-5L index, DLQI, ABSIS scores. Among the four variables the following six paths were hypothesized and tested: (1) ABSIS → DLQI (+); (2) ABSIS → EQ-5D-5L (−); (3) ABSIS → SWLS (−); (4) DLQI → EQ-5D-5L (−); (5) DLQI → SWLS (−) and (6) EQ-5D-5L → SWLS (+). To determine whether the expected model was plausible with the data, we used a Chi-square test statistic. The following goodness-of-fit indices were used based on Hu and Bentler’s recommendations: comparative fit index (CFI), root mean square error of approximation (RMSEA), and standardized root mean square residual (SRMR) [38]. A CFI of > 0.95 was considered as an indicative of good fit. Acceptable threshold values of RMSEA and SRMR were ≤ 0.06 and ≤ 0.08, respectively. *p* values less than 0.05 were taken as statistically significant. The path analysis was conducted using AMOS 23, all other analyses were performed using SPSS 25.0 (Armonk, NY: IBM Corp.).

## Results

### General characteristics

A total of 77 patients completed the SWLS, DLQI and EQ-5D-5L questionnaires. Characteristics of the patient population are presented in Table 1. Mean age was 55.4 (SD 14.8) years, and 58% were female. The majority of patients (73%) were diagnosed with pemphigus vulgaris and 26% with pemphigus foliaceus. One patient had IgA pemphigus. Mean ABSIS score of the study population was 11.7 (SD 17.3). Based on ABSIS, 37 (48%), 20 (26%), 16 (21%) and 4 (5%) patients were grouped into mild, moderate and significant and extensive pemphigus subgroups. Mean EQ-5D-5L index and EQ VAS scores were 0.84 (0.22) and 69 (22), respectively.

**Table 1:** General characteristics of the sample

Characteristics	*N* (%) or mean (SD)
Number of subjects	77
Age (years)	55.4 (14.8)
Female	45 (58.4%)
Education	
Primary school	13 (16.9%)
High school	42 (54.5%)
College/university	22 (28.6%)
Employment^a^	
Employed full-time	29 (37.7%)
Employed part-time	8 (10.4%)
Unemployed	4 (5.2%)
Disability pensioner	12 (15.6%)
Retired	27 (35.1%)
Student	1 (1.3%)
Other	1 (1.3%)
Disease duration (years)	4.4 (5.3)
Type of pemphigus	
Pemphigus vulgaris	56 (72.7%)
Pemphigus foliaceus	20 (26.0%)
IgA pemphigus	1 (1.3%)
ABSIS (0–206)	12.6 (18.9%)
Severity groups	
Limited (ABSIS 0–3)	37 (48.1%)
Moderate (ABSIS 4–16)	20 (26.0%)
Significant (ABSIS 17–52)	16 (20.8%)
Extensive (ABSIS 53–206)	4 (5.2%)
DLQI (0–30)	5.36 (6.88)
EQ-5D-5L (–0.285–1)	0.84 (0.22)
Current treatment	
None	2 (2.6%)
Topical therapy (only)	7 (9.1%)
Systemic therapy^a^	68 (88.3%)

^a^Combinations are possible

*ABSIS* Autoimmune Bullous Skin Disorder Intensity Score, DLQI Dermatology Life Quality Index, *SWLS* Satisfaction with Life Scale

### Descriptive statistics of SWLS

**Fig. 1:**
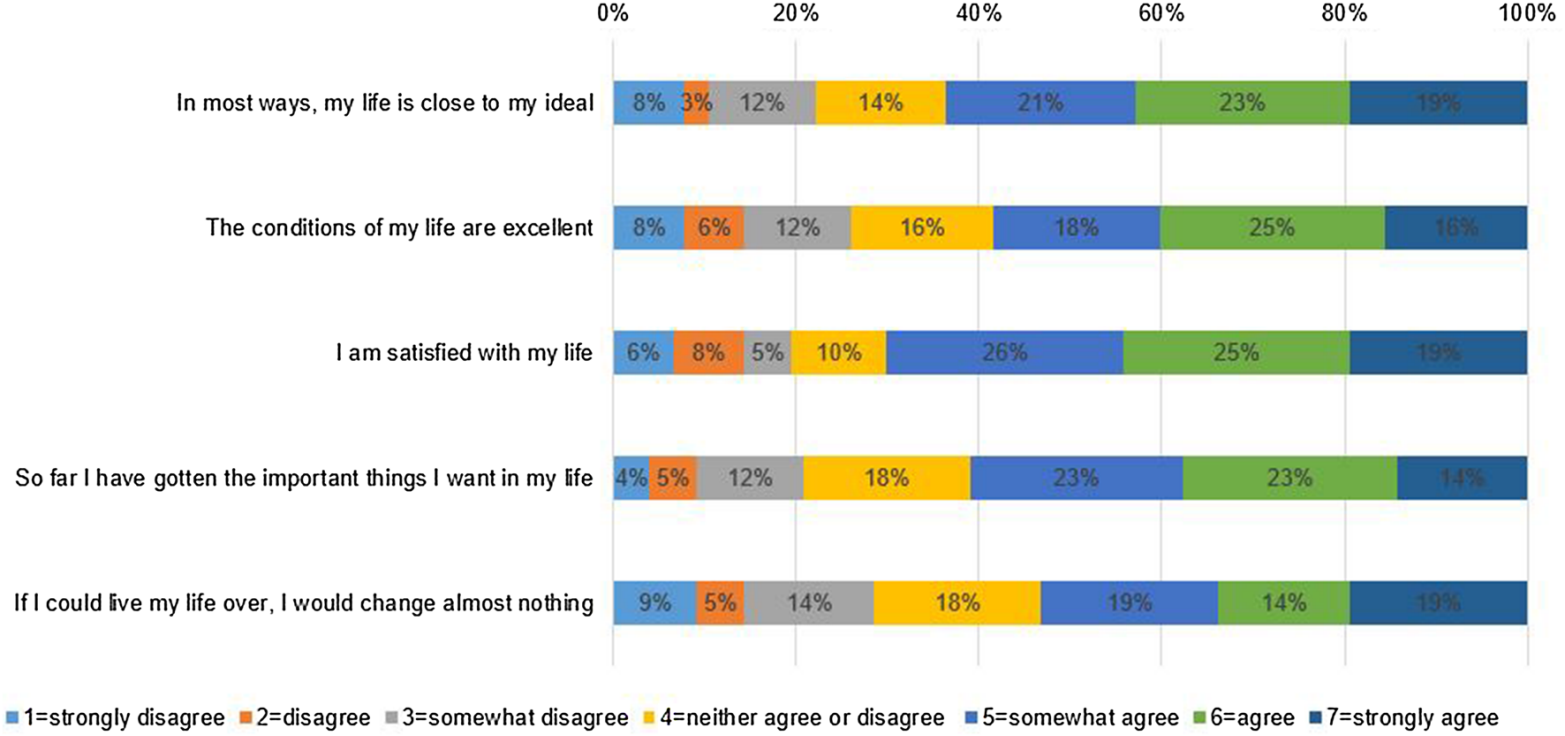
Distribution of the responses on the five items of SWLS*. SWLS* Satisfaction with Life Scale

Distribution of responses on the five items of SWLS is presented in Fig. 1. The highest rate of ‘strongly agree’ responses was found for items 1, 3 and 5 (19%), while the highest rate of ‘strongly disagree’ responses was observed for item 5. The mean SWLS total score in the sample was 4.76 (SD 1.52). All five items exhibited very similar means: item 1: “In most ways, my life is close to my ideal” (4.83, SD 1.76), item 2: “The conditions of my life are excellent” (4.63, SD 1.80), item 3: “I am satisfied with my life” (4.92, SD 1.77), item 4:”So far I have gotten the important things I want in my life” (4.7, SD 1.58) and item 5: “If I could live my life over, I would change almost nothing” (4.55, SD 1.85).

Six (7.8%) patients indicated extreme dissatisfaction, five (6.5%) dissatisfaction, 14 (18.2%) slightly below average in life satisfaction, 16 (20.8%) average satisfaction, 21 (27.3%) high satisfaction and 15 (19.5%) very high satisfaction with their lives, respectively.

### Correlation analysis

Table 2 reports the correlations among ABSIS, DLQI, EQ-5D-5L and SWLS total scores. Moderate correlations were found between ABSIS and DLQI (*r* = 0.425, *p* < 0.001), between DLQI and EQ-5D-5L (*r* = − 0.593, *p* < 0.001) and between EQ-5D-5L and SWLS (*r* = 0.465, *p* = 0.011). ABSIS demonstrated a weak correlation with EQ-5D-5L (*r* = − 0.345, *p* = 0.002) and with SWLS (*r* = − 0.237, *p* = 0.038). A weak correlation was detected between DLQI and SWLS (*r* = − 0.288, *p* = 0.011).

**Table 2:** Correlation matrix of the outcome measures

	ABSIS	DLQI	EQ-5D-5L
ABSIS (0–206)	–	–	–
DLQI (0–30)	0.425 (*p* < 0.001)	–	–
EQ-5D-5L (–0.285–1)	− 0.345 (*p* = 0.002)	− 0.593 (*p* < 0.001)	–
SWLS (1–7)	− 0.237 (*p* = 0.038)	− 0.288 (*p* = 0.011)	0.465 (*p* = 0.011)

For ABSIS and DLQI, higher scores represent worse health status and for EQ-5D-5L and SWLS higher scores refer to better health status/satisfaction with life

*ABSIS* Autoimmune Bullous Skin Disorder Intensity Score, *DLQI* Dermatology Life Quality Index, *SWLS* Satisfaction with Life Scale

### Subgroup analysis of SWLS

Table 3 presents the total SWLS scores in subgroups of patients based on gender, age, education and type of pemphigus. Slightly higher mean SWLS scores were observed in females (4.82), patents aged > 65 (4.91), those with a college or university degree (5.05) and pemphigus foliaceus patients (4.93); however, the differences were insignificant.

**Table 3:** SWLS scores in subgroups of patients

	Mean (SD)	Median (IQR)	*p* value
Gender			0.688
Male	4.68 (1.34)	4.90 (2.05)	
Female	4.82 (1.65)	5.20 (2.40)	
Age groups			0.181
18–45	5.22 (1.45)	5.3 (1.8)	
46–65	4.45 (1.59)	4.7 (2.2)	
66–	4.91 (1.39)	5.2 (2.3)	
Education			0.577
Primary	4.58 (2.11)	4.80 (4.00)	
Secondary	4.66 (1.46)	4.80 (2.20)	
Tertiary	5.05 (1.25)	5.20 (1.75)	
Type of pemphigus			0.562
Pemphigus vulgaris	4.70 (1.59)	5.00 (2.20)	
Pemphigus foliaceus	4.93 (1.34)	5.00 (2.20)	

*SWLS* Satisfaction with Life Scale

### Results of the path analysis model

**Fig. 2:**
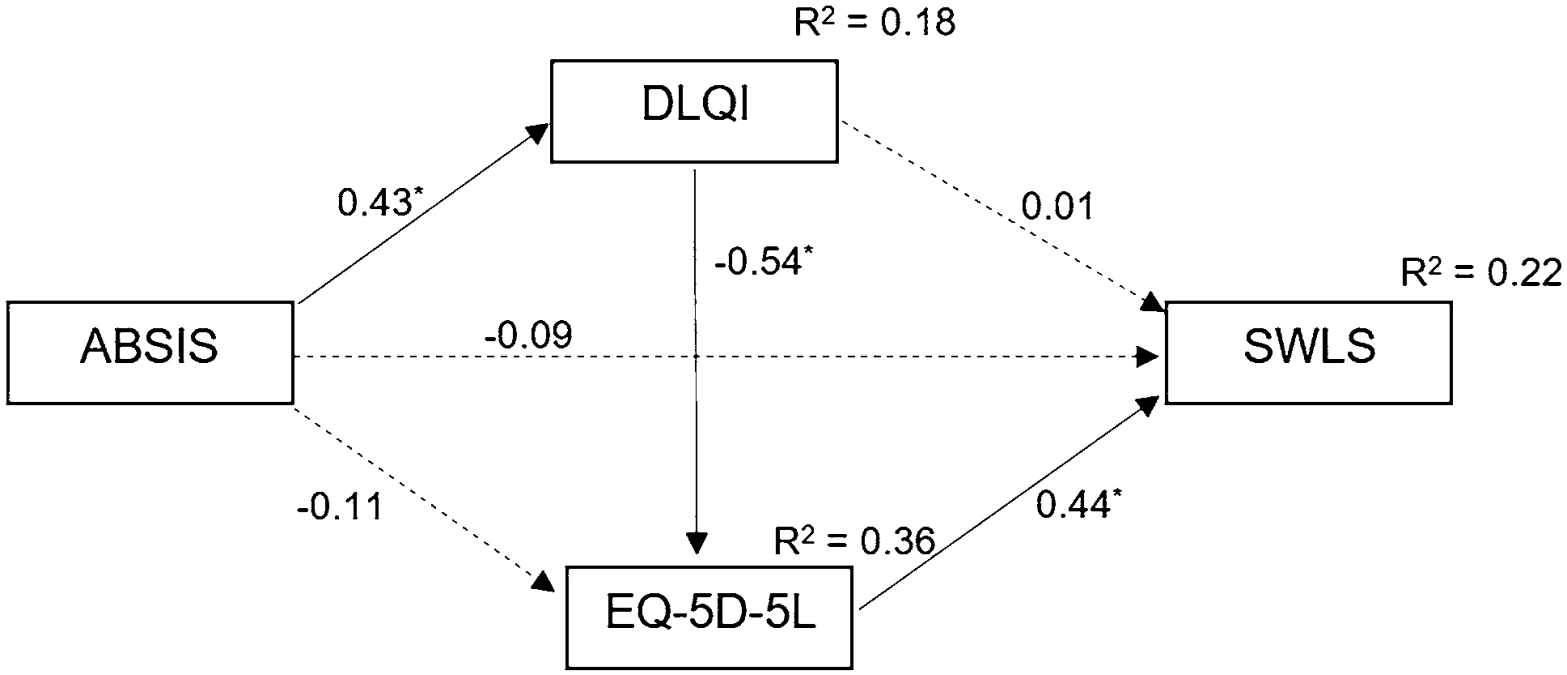
Path model. Notes: every path coefficient is standardized (*p < 0.001). Dashed lines represent insignificant paths. *ABSIS* Autoimmune Bullous Skin Disorder Intensity Score, *DLQI* Dermatology Life Quality Index, *SWLS* Satisfaction with Life Scale

The path analysis model we evaluated is depicted in Fig. 2. The results indicate that there was a statistically significant path from ABSIS → DLQI → EQ-5D-5L → SWLS. The model showed overall good fit indices: Chi-square = 0.00 CFI = 1.00, SRMR = 0.00. The model explained 18.0% of the variance of DLQI, 36.2% of the EQ-5D-5L and 22.3% of the SWLS.

ABSIS had a positive effect on DLQI (*β* = 0.425; *p* < 0.001) indicating that the disease severity assessed by the dermatologist affects the skin-specific HRQoL perceived by the patient. The ABSIS scores demonstrated no direct impact on EQ-5D-5L index scores (*β* = − 0.114; *p* = 0.262). An indirect path was detected between ABSIS and EQ-5D-5L index through DLQI score. Contrary to our expectations, there was no direct relationship between ABSIS and SWLS (*β* = − 0.090; *p* = 0.428). DLQI had a negative effect on EQ-5D-5L (*β* = − 0.545; *p* < 0.001) implying that skin-specific HRQoL did influence general HRQoL. There was no direct relationship between DLQI and SWLS (*β* = 0.011; *p* = 0.935). However, an indirect patch was detected by which the DLQI affected SWLS through the EQ-5D-5L. Finally, a significant relationship was found between EQ-5D-5L and SWLS (*β* = 0.440; *p* < 0.001).

## Discussion

The aim of the present study was to investigate life satisfaction as a proxy for subjective well-being and its predictors in pemphigus patients through a path analysis. The mean SWLS score was 4.76 indicating an overall average satisfaction with life. Nearly one-third (32.3%) of patients rated their satisfaction with their life to be ‘below average’. Life satisfaction was independent from patients’ age, gender, level of education and type of disease. A path analysis revealed the following indirect path: ABSIS → DLQI → EQ-5D-5L → SWLS. Physician’s objective assessment of disease severity (ABSIS) had no direct impact on life satisfaction. This route highlights the mediating effect of the patients’ perception about HRQoL on satisfaction with life.

Coping with the daily limitations caused by a chronic disease often affects patients’ perception of health and satisfaction with life [39]. Previous research has shown significantly lower life satisfaction in patients with various chronic skin diseases [19–25]. For example, 16.6% of patients with atopic dermatitis in the US reported being at least somewhat dissatisfied with life [25]. In our study, 14.3% of patients declared to be dissatisfied or highly dissatisfied. A potential explanation for this could be that the majority of patients were well-treated and indicated a relatively low disease severity and less deterioration in their HRQoL, which was very likely responsible for the rather positive results regarding life satisfaction.

Earlier research suggests a good validity of the EQ-5D-5L in pemphigus patients [8]. Nevertheless, it may fail to capture some of the broader impact of pemphigus on HRQoL and subjective well-being [11]. For example, in a recent survey among members of the general population in the UK, respondents identified several important aspects of health that are not covered by the five dimensions of the EQ-5D, such as mental and emotional health and happiness [40]. Non-health outcomes including ability to work, financial security and social support in one’s life might be important determinants of life satisfaction.

A practical implication of our study is that objective perception of the physician on disease severity only has an indirect effect on satisfaction with life. In routine practice, disease severity and HRQoL instruments regularly used to assess patients’ health status may be complemented with a measure of subjective well-being, such as SWLS to achieve a more holistic assessment of patients’ lives and optimise pemphigus care. This would also enable the use of subjective well-being outcomes to aid health policy decisions.

This is the first study in the literature to report on life satisfaction in pemphigus patients. Furthermore, our analyses were based on a relatively large number of patients, considering the rarity of the disease. Among the study’s limitations, it needs to be acknowledged that the study was conducted in university dermatology departments, and systematic selection bias due to centre effects could have been present. Thus, the results may not be representative of the entire population of patients with pemphigus. However, the study population included a large variety of pemphigus patients in terms of clinical characteristics and severity. Second, many other factors possibly influencing life satisfaction have not been measured in this survey. Further research is required to identify unexplored the drivers of subjective well-being in pemphigus patients.

In conclusion, an overall average satisfaction with life was identified in patients with pemphigus. A higher level of general HRQoL is the major determinant of life satisfaction in pemphigus patients. The path analysis identified skin-specific HRQoL and disease severity as indirect predictors of satisfaction with life. These results underscore the importance of achieving remission in benefiting the lives of patients with pemphigus.

## References

[1] Kershenovich R, Hodak E, Mimouni D (2014). Diagnosis and classification of pemphigus and bullous pemphigoid. Autoimmun. Rev..

[2] Kridin K, Zelber-Sagi S, Bergman R (2017). Pemphigus vulgaris and pemphigus foliaceus: differences in epidemiology and mortality. Acta Derm. Venereol..

[3] Meyer N, Misery L (2010). Geoepidemiologic considerations of auto-immune pemphigus. Autoimmun. Rev..

[4] Rencz F, Brodszky V, Stalmeier PF, Tamasi B, Karpati S, Pentek M, Baji P, Mitev AZ, Gulacsi L (2016). Valuation of pemphigus vulgaris and pemphigus foliaceus health states: a convenience sample experiment. Br. J. Dermatol..

[5] Rencz F, Gulacsi L, Tamasi B, Karpati S, Pentek M, Baji P, Brodszky V (2015). Health-related quality of life and its determinants in pemphigus: a systematic review and meta-analysis. Br. J. Dermatol..

[6] Wang EQ, Radjenovic M, Castrillon MA, Feng GHY, Murrell DF (2018). The effect of autoimmune blistering diseases on work productivity. J. Eur. Acad. Dermatol. Venereol..

[7] Sebaratnam DF, McMillan JR, Werth VP, Murrell DF (2012). Quality of life in patients with bullous dermatoses. Clin. Dermatol..

[8] Tamasi B, Brodszky V, Pentek M, Gulacsi L, Hajdu K, Sardy M, Szegedi A, Bata-Csorgo Z, Kinyo A, Rencz F (2019). Validity of the EQ-5D in patients with pemphigus vulgaris and pemphigus foliaceus. Br. J. Dermatol..

[9] Rencz F, Gulacsi L, Tamasi B, Karpati S, Brodszky V (2015). Social utility values for pemphigus vulgaris and foliaceus: a composite time trade-off study. Value Health.

[10] Diener E, Lucas RE, Oishi S (2018). Advances and open questions in the science of subjective well-being. Collabra Psychol..

[11] Cubi-Molla P, de Vries J, Devlin N (2014). A study of the relationship between health and subjective well-being in Parkinson’s disease patients. Value Health.

[12] Lamu AN, Olsen JA (2016). The relative importance of health, income and social relations for subjective well-being: an integrative analysis. Soc. Sci. Med..

[13] Vázquez C, Rahona J, Gómez D, Caballero F, Hervás G (2015). A national representative study of the relative impact of physical and psychological problems on life satisfaction. J. Happiness Stud..

[14] Korman, N.J., Zhao, Y., Pike, J., Roberts, J., Sullivan, E.: Patient satisfaction with current psoriasis treatment: a real-world study in the USA. Dermatol Online J. 10.1177/247553031600100207 10.1177/247553031600100207 27267186

[15] Ayala F, Sampogna F, Romano GV, Merolla R, Guida G, Gualberti G, Paparatti UD, Amerio P, Balato N, Potenza C, Daniele Study, G. (2014). The impact of psoriasis on work-related problems: a multicenter cross-sectional survey. J. Eur. Acad. Dermatol. Venereol..

[16] Claudepierre P, Lahfa M, Levy P, Barnetche T, Bonnet I, Aubert R, Roquelaure Y (2018). The impact of psoriasis on professional life: PsoPRO, a French National Survey. J. Eur. Acad. Dermatol. Venereol..

[17] Theut Riis P, Thorlacius L, Knudsen List E, Jemec GBE (2017). A pilot study of unemployment in patients with hidradenitis suppurativa in Denmark. Br. J. Dermatol..

[18] Boncz I, Sebestyen A (2006). Financial deficits in the health services of the UK and Hungary. Lancet.

[19] Kulczycka L, Sysa-Jedrzejowska A, Robak E (2009). Life satisfaction together with positive and negative aspects in Polish patients with systemic lupus erythematosus. J. Eur. Acad. Dermatol. Venereol..

[20] Solovan C, Marcu M, Chiticariu E (2014). Life satisfaction and beliefs about self and the world in patients with psoriasis: a brief assessment. Eur. J. Dermatol..

[21] Bonnaud-Antignac A, Bourdon M, Dreno B, Quereux G (2017). Coping strategies at the time of diagnosis and quality of life 2 years later: a Study in Primary Cutaneous Melanoma Patients. Cancer Nurs..

[22] McLellan C, Sisic M, Oon HH, Tan J (2018). Preliminary validation of the HS-QoL: a quality-of-life measure for hidradenitis suppurativa. J. Cutan. Med. Surg..

[23] Zelic SB, Rubesa G, Brajac I, Peitl MV, Pavlovic E (2016). Satisfaction with life and coping skills in the acute and chronic urticaria. Psychiatr. Danub.

[24] Basinska MA, Drozdowska M (2013). Emotional intelligence as an indicator of satisfaction with life of patients with psoriasis. Postepy Dermatol. Alergol..

[25] Silverberg JI, Gelfand JM, Margolis DJ, Boguniewicz M, Fonacier L, Grayson MH, Simpson EL, Ong PY, Chiesa Fuxench ZC (2018). Patient burden and quality of life in atopic dermatitis in US adults: a population-based cross-sectional study. Ann. Allergy Asthma Immunol..

[26] Rencz F, Gulacsi L, Drummond M, Golicki D, Prevolnik Rupel V, Simon J, Stolk EA, Brodszky V, Baji P, Zavada J, Petrova G, Rotar A, Pentek M (2016). EQ-5D in Central and Eastern Europe: 2000–2015. Qual. Life Res..

[27] EuroQol G (1990). EuroQol–a new facility for the measurement of health-related quality of life. Health Policy.

[28] Devlin NJ, Shah KK, Feng Y, Mulhern B, van Hout B (2018). Valuing health-related quality of life: an EQ-5D-5L value set for England. Health Econ..

[29] Finlay AY, Khan GK (1994). Dermatology Life Quality Index (DLQI)—a simple practical measure for routine clinical use. Clin. Exp. Dermatol..

[30] Rencz F, Gulacsi L, Pentek M, Poor AK, Sardy M, Hollo P, Szegedi A, Remenyik E, Brodszky V (2018). Proposal of a new scoring formula for the Dermatology Life Quality Index in psoriasis. Br. J. Dermatol..

[31] Diener E, Emmons RA, Larsen RJ, Griffin S (1985). The satisfaction with Life Scale. J. Pers. Assess..

[32] Martos T, Sallay V, Désfalvi J, Szabó T, Ittzés A (2014). Psychometric characteristics of the Hungarian version of the Satisfaction with Life Scale (SWLS-H). Mentálhigiéné és Pszichoszomatika.

[33] Diener, E.: Understanding Scores on the Satisfaction with Life Scale (2006). http://labs.psychology.illinois.edu/~ediener/Documents/Understanding%20SWLS%20Scores.pdf. Accessed 15 March 2019

[34] Pfutze M, Niedermeier A, Hertl M, Eming R (2007). Introducing a novel Autoimmune Bullous Skin Disorder Intensity Score (ABSIS) in pemphigus. Eur. J. Dermatol..

[35] Daniel BS, Hertl M, Werth VP, Eming R, Murrell DF (2012). Severity score indexes for blistering diseases. Clin. Dermatol..

[36] Hanna S, Kim M, Murrell DF (2016). Validation studies of outcome measures in pemphigus. Int. J. Womens Dermatol..

[37] Boulard C, Duvert Lehembre S, Picard-Dahan C, Kern JS, Zambruno G, Feliciani C, Marinovic B, Vabres P, Borradori L, Prost-Squarcioni C, Labeille B, Richard MA, Ingen-Housz-Oro S, Houivet E, Werth VP, Murrell DF, Hertl M, Benichou J, Joly P, International Pemphigus Study, G. (2016). Calculation of cut-off values based on the Autoimmune Bullous Skin Disorder Intensity Score (ABSIS) and Pemphigus Disease Area Index (PDAI) pemphigus scoring systems for defining moderate, significant and extensive types of pemphigus. Br. J. Dermatol..

[38] Hu LT, Bentler PM (1999). Cutoff criteria for fit indexes in covariance structure analysis: conventional criteria versus new alternatives. Struct. Equ. Model. Multidiscip. J..

[39] Testa MA, Simonson DC (1996). Assessment of quality-of-life outcomes. N. Engl. J. Med..

[40] Shah KK, Mulhern B, Longworth L, Janssen MF (2017). Views of the UK general public on important aspects of health not captured by EQ-5D. Patient.

